# Computer 3D modeling of radiofrequency ablation of atypical cartilaginous tumours in long bones using finite element methods and real patient anatomy

**DOI:** 10.1186/s41747-022-00271-3

**Published:** 2022-04-28

**Authors:** Ricardo Rivas Loya, Paul C. Jutte, Thomas C. Kwee, Peter M. A. van Ooijen

**Affiliations:** 1 Department of Radiotherapy, University of Groningen, University Medical Center Groningen, Hanzeplein 1, 9713 GZ Groningen, The Netherlands; 2 Department of Orthopedics, University of Groningen, University Medical Center Groningen, Groningen, The Netherlands; 3 Department of Radiology, University of Groningen, University Medical Center Groningen, Groningen, The Netherlands

**Keywords:** Bone neoplasms, Bone and bones, Catheter ablation (radiofrequency), Computer simulation, Finite element analysis

## Abstract

**Background:**

Radiofrequency ablation (RFA) is a minimally invasive technique used for the treatment of neoplasms, with a growing interest in the treatment of bone tumours. However, the lack of data concerning the size of the resulting ablation zones in RFA of bone tumours makes prospective planning challenging, needed for safe and effective treatment.

**Methods:**

Using retrospective computed tomography and magnetic resonance imaging data from patients treated with RFA of atypical cartilaginous tumours (ACTs), the bone, tumours, and final position of the RFA electrode were segmented from the medical images and used in finite element models to simulate RFA. Tissue parameters were optimised, and boundary conditions were defined to mimic the clinical scenario. The resulting ablation diameters from postoperative images were then measured and compared to the ones from the simulations, and the error between them was calculated.

**Results:**

Seven cases had all the information required to create the finite element models. The resulting median error (in all three directions) was -1 mm, with interquartile ranges from -3 to 3 mm. The three-dimensional models showed that the thermal damage concentrates close to the cortical wall in the first minutes and then becomes more evenly distributed.

**Conclusions:**

Computer simulations can predict the ablation diameters with acceptable accuracy and may thus be utilised for patient planning. This could allow interventional radiologists to accurately define the time, electrode length, and position required to treat ACTs with RFA and make adjustments as needed to guarantee total tumour destruction while sparing as much healthy tissue as possible.

## Key points

- Radiofrequency ablation is a technique with great potential to treat bone neoplasms.
- There is, however, little information regarding the expected outcomes, making planning challenging.
- Computer models using the finite element method could be used to simulate the interventions.
- Computer simulations could help planning safe and effective interventions.

## Background

Radiofrequency ablation (RFA) is a minimally invasive technique that has been commonly used for the treatment of neoplasms in the liver, kidney, adrenal glands, bone, lung, and breast [1]. Its use for the treatment of bone tumours has been mostly focused on osteoid osteoma, but there has been an interest in the treatment of other types of bone tumours, particularly the ones in the benign spectrum, such as chondroblastoma [2–6] and osteoblastoma [7–9], but also atypical cartilaginous tumours (ACTs) [10, 11]. Due to its minimal invasiveness, in contrast to more aggressive treatments such as open surgery, it allows for shorter recovery times, targeted tumour destruction, and low complication rates [12].

To guarantee optimal treatment, *i.e.*, total tumour destruction while minimising damage to healthy tissue [1], it is important to know the extension of the ablation zone. There is, however, little to no data concerning the size of the ablation zone for RFA of bone tumours, particularly for tumours other and larger than osteoid osteoma (*i.e.*, > 2 cm). Given the interest of our research group to expand the use of RFA from osteoid osteoma to other tumours such as ACTs, accurate and reliable planning are needed to guarantee safe and effective tumour ablations. In a previous initial study by our team in patients with an ACT in which the resulting ablation diameters after RFA treatment were measured, it was observed that the ablation zones grew larger than expected, particularly in contrast to those after RFA in other tissues, such as the liver and kidney [11]. Similar findings were observed by Neeman et al. [13], who performed RFA of a chordoma and suggested the larger than expected ablation volume may be due to the high-water content of the tumour and its relatively poor vascularity, resulting in higher electrical and thermal conductivities than that of liver or kidney tissue.

To further understand the effects of the bone and tumour tissue on the resulting ablation zone, a previous and yet to be published study by our research group used computer models with the finite element method (FEM) to simulate the RFA of ACT. The implementation used a simplified two-dimensional (2D) geometry of the patient and a fractional factorial analysis to determine optimal parameters to replicate clinical cases with the computer model. These models had limitations, as they did not take into account the full three-dimensional (3D) characteristics of the anatomic location of interest. To further test, the validity of these assumptions and in an attempt to develop an accurate patient-specific planning system for clinical use in RFA of ACTs, this study aimed to develop 3D models of the patient’s anatomy and test the computational model’s accuracy in replicating the ablation zones seen in clinical practice.

## Methods

This study consisted of the following steps:

1. Collection, segmentation, and manual registration of preprocedural and intraprocedural images of cases of ACT that were treated with RFA;
2. Collection of the relevant clinical parameters of the intervention, such as the size of the active RFA electrode (either 2- or 3-cm long) and the amount of time the energy was applied;
3. Generation of standard triangle language (STL) files from the segmented images which were then transformed into 3D meshes;
4. Application of a FEM utilising the 3D patient-specific models from patient data;
5. Measurement of the ablation diameters on post-RFA magnetic resonance (MR) images and the computer models.

### Image segmentation and registration

Cases from a previous study [11] of patients that had undergone computed tomography (CT)-guided RFA of ACT were used to create the patient-specific meshes. Only patients that agreed and signed a written form at the time of the intervention about the potential use of their anonymised data for scientific research were included in the study. Only cases in which it was possible to determine the final position of the RFA electrode from the intra-procedural CT images and in which it was possible to properly segment the tumour and surrounding bone were chosen. The pre-RFA MR images (were used to segment the tumour, because tumour boundaries are usually not clearly visible on CT). The MR images were acquired using a 1.5 T MRI scanner (Siemens, Erlangen, Germany) with a surface coil. Fat-suppressed Short Tau Inversion Recovery (STIR) T2-weighted sequences (TR/TE/TI: 8270/160/19 ms, 4-mm-slice thickness) and T1-weighted images (TR/TE: 500/19 ms, 4-mm-slice thickness) were acquired before and after the administration of an intravenous gadolinium-based contrast agent (0.1-mmol gadoterate meglumine (Dotarem®; Guerbet) per kilogram of body weight). As part of the routine MRI protocol, the images were acquired in two planes (transversal and either coronal or sagittal). The intraprocedural CT images (Somaton Definition AS, Siemens Medical Systems, Erlangen, Germany; 100 kVp, 49 mAs, 0.8 mm pitch) were used to segment the bones and the electrode.

All segmentations were done with Materialize Mimics (https://www.materialise.com) by the first author and later assessed by a musculoskeletal radiologist to confirm their accuracy. The segmentations were exported to STL format and Gmsh [14] (https://gmsh.info), and a 3D finite element mesh generator was used to generate the 3D meshes using the STL files. Because of the artifacts caused by the electrode on the CT data, the segmented electrode was only used to find the location of the electrode and then it was replaced by a modeled electrode, generated on Gmsh, replicating its dimensions. Gmsh was also used to create a mesh for the muscle tissue, which was assumed as a large cube surrounding the cortical bone. The surface of the trabecular bone was determined by applying an offset of -2.5 mm on the surface of the segmented cortical bone.

Using MeshLab [15] (https://www.meshlab.net), meshes of multiple characteristic lengths were created and a mesh convergence analysis was done for each of the models, with the electrode having the smallest triangles (0.1−0.25 mm) and the muscles having much larger triangles. With Autodesk Meshmixer (http://www.meshmixer.com), manual refinement and reduction of triangles were performed, reducing the triangles in sections of the bone far away from the electrode and increasing them close to the tumour and electrode. All models were defeatured, removing fine details to reduce the tetrahedron needed and thus also reduce the computational requirements.

### Clinical parameters

The radiologist who performed the RFA registered the amount of time with the electrode at the desired target temperature of 90 °C and also indicated the amount of time with the applicator at a temperature higher than 60 °C. The length of the active electrode used during each intervention was also registered, which was either 2 or 3 cm.

A musculoskeletal radiologist with 10 years of experience measured the ablation diameters in three orthogonal directions on the post-RFA MR images (same sequences used before RFA) that were acquired 3 months after the intervention. The results from the simulations were measured by the first author, with 5 years of experience in image processing, and who ran the computer simulations. The results were measured in three orthogonal directions as to match the measurements by the radiologist, and both results were compared. Note that the first author was blinded to the radiologist’s measurements and vice versa. The measurements were labeled as “longest,” “shortest,” and “other” ablation diameters, with the longest diameter being the diameter along the electrode axis and the other two orthogonal to it.

### Finite element model

To determine the thermal damage over time of RFA of ACT with a non-cooled temperature-controlled ablation protocol with a control temperature of 90 °C, finite element models were created, in line with a previous, yet to be published, a study from our research group. The implementation was done in FEniCS, a solver for finite element problems [16].

### Equations governing radiofrequency ablation

In RFA, an alternating current of approximately 500 kHz is applied with an electrode into a target tissue. The current induces heating in the tissues surrounding the electrode, causing thermal damage to cells exposed to high temperatures. To simulate RFA, a coupled electric-thermal problem has then to be solved, where tissue heating is due to Joule heating. Additionally, since the thermal damage is also dependent on the time exposed to a given temperature, a cell death model has to be utilised to relate temperature, time, and the robustness of the tissues to thermal damage.

For the electrical problem, a quasi-static approach can be taken, as the tissues can be seen as totally resistive to the current in the RF range [17]. The heat source, Q_RF_, is given by:

1 $$ {Q}_RF={\sigma}_i(T){\left|E\right|}^2 $$

where σ_*i*_ is the temperature-dependent electrical conductivity (S/m) of each tissue and **E** is the electric field intensity in (V/m), which is then defined by the Laplace equation, where *V* is the root mean squared value of the applied voltage *V*:

2 $$ \nabla \cdotp {\upsigma}_i\left(\mathrm{T}\right)\nabla \mathrm{V}=0 $$

The RFA procedure used for these patients was a temperature-controlled procedure, in which the voltage is automatically regulated by the RFA machine to maintain a predefined target control temperature, set by the interventional radiologist. To mimic that, a proportional-integral controller can be used, defining the root mean square (RMS) voltage (V) applied at the electrode boundary, V_RMS._, as follows:

3 $$ {\mathrm{V}}_{RMS.}={\mathrm{K}}_{\mathrm{p}}\left({\mathrm{T}}_{\mathrm{target}}-\mathrm{T}\left(\mathrm{t}\right)\right)+{\mathrm{K}}_{\mathrm{i}}\int_0^{\tau}\left({\mathrm{T}}_{\mathrm{target}}-\mathrm{T}\left(\mathrm{t}\right)\right)\ \mathrm{dt} $$

For the controller, the initial voltage is set to zero and then modulated as a function of the difference between the target temperature T_target_ and the temperature at the electrode’s tip T(t) at the time t, the error proportionally constant K_p_, and the integral proportionality constant K_i_. These constants are model dependent, but values of *K*_*p*_ = 1.15 V/K and *K*_*i*_ = 0.06 V/K/s have been found to be a good approximation for *ex vivo* bone RFA [18].

The temperature-dependent properties of the electrical conductivity can be modeled with a piece-wise function [19]. First, there is a linear increase of 1.5%/°C until the point of tissue vaporisation, where then a sudden drop in the electrical conductivity occurs, defined as follows:

4 $$ {\sigma}_i\left(\mathrm{T}\right)=\left\{\begin{array}{ccc}{\sigma}_i+\varDelta {\sigma}_i\left(T-37{}^{\circ}\mathrm{C}\right),& \mathrm{T}\le 100{}^{\circ}\mathrm{C}& \\ {}{\sigma}_{100{}^{\circ}\mathrm{C}}+\left({\sigma}_{\mathrm{vap}}-{\sigma}_{100{}^{\circ}\mathrm{C}}\right)\frac{\left(\mathrm{T}-100{}^{\circ}\mathrm{C}\right)}{5},& 100{}^{\circ}\mathrm{C}<\mathrm{T}\le 105{}^{\circ}\mathrm{C}& \\ {}{\sigma}_{\mathrm{vap}},& \mathrm{T}>105{}^{\circ}\mathrm{C}& \end{array}\right\} $$

where ∆*σ*_*i*_ corresponds to the 1.5% linear increase in the electrical conductivity per °C from the baseline electrical conductivity, *σ*_*i*_, at 37 °C. *σ*_vap_ is the electrical conductivity of vaporised tissue, with a value of *σ*_vap_ = 10*x*10^−3^ (S/m) [19].

The thermal problem is governed by Penne’s bioheat equation [20], which is a modified version of the heat equation. If tissues get close to the boiling point, tissue vaporisation occurs, which has important effects on both the electrical and thermal problems. Additionally, to account for the phase change into vapor, the enthalpy method [21] is utilised:

$$ \frac{\partial h}{\mathrm{\partial t}}=\nabla \cdotp \left({k}_i\left(\mathrm{T}\right)\nabla \mathrm{T}\right)+{Q}_{\mathrm{RF}}-{Q}_p $$

where *h* is the enthalpy, which is a piece-wise function that relates the density and specific heat to temperature and accounts for phase change at high temperatures, *k*_*i*_ is the temperature-dependent thermal conductivity (W/m ∙ K), *T* is the temperature, *Q*_RF_ the heat source (W/*m*^3^), and *Q*_*p*_ is the blood perfusion heat loss (W /*m*^3^), defined as follows:

5 $$ {\mathrm{Q}}_{\mathrm{p}}={\upomega}_i\left(\Omega \right){\uprho}_{\mathrm{b}}{\mathrm{c}}_{\mathrm{b}}\left[\mathrm{T}-{\mathrm{T}}_{\mathrm{b}}\right] $$

where ω_*i*_ is a tissue-dependent perfusion coefficient (*s*^−1^) that depends on the cell viability Ω, from the cell death model, and indicates whether a cell is alive (with blood perfusion) or death (without blood perfusion). ρ_b_ is the density of the blood (kg/*m*^3^), c_b_ the specific heat of the blood (J/kg ∙ K), and *T*_*b*_ the temperature of the blood (K).

To account for the temperature dependency of the heat capacity, a linear increase is first considered, followed by a sudden change in heat capacity at boiling temperatures, modeled using the enthalpy method [19, 21]:

6 $$ h=\left\{\begin{array}{ccc}{\rho}_i{c}_i\left(T-37{}^{\circ}\mathrm{C}\right),& 37{}^{\circ}\mathrm{C}\le \kern0.5em T\le 99{}^{\circ}\mathrm{C}& \\ {}h(99)+{h}_{\mathrm{fg}}{\mathrm{C}}_i\ \frac{\left(T-99{}^{\circ}\mathrm{C}\right)}{\left(100{}^{\circ}\mathrm{C}-99{}^{\circ}\mathrm{C}\right)},& 99{}^{\circ}\mathrm{C}<T\le 100{}^{\circ}\mathrm{C}& \\ {}h(100)+{\rho}_{\mathrm{vap}}{c}_{\mathrm{vap}}\left(T-100{}^{\circ}\mathrm{C}\right),& T>100{}^{\circ}\mathrm{C}& \end{array}\right\} $$

where *ρ*_*i*_c_*i*_ are the baseline values of density $\Big(\frac{kg}{m^3}$) and specific heat $\left(\frac{J}{kgK}\right)$ of each tissue, *h*_fg_is the latent heat of vaporisation (2.25x${10}^6\frac{J}{kg}$), C_*i*_ is the water fraction of each tissue, and *ρ*_vap_(370 $\frac{kg}{m^3}$) and *c*_vap_ (2156 $\frac{J}{kgK}\Big)$ are the density and specific heat of the vaporised tissue [19].

For the temperature dependence of the thermal conductivity, *k*_*i*_, of each tissue, a linear increase (∆*k*_*i*_) of 0.33% per degree Celsius was considered until the point of vaporisation, at which a maximum value was set [22, 23], as defined as follows:

7 $$ {k}_i\left(\mathrm{T}\right)=\left\{\begin{array}{cc}{k}_i+\varDelta {k}_i\left(T-37{}^{\circ}\mathrm{C}\right),& T\le 100{}^{\circ}\mathrm{C}\\ {}{k}_i+\varDelta {k}_i\left(100{}^{\circ}\mathrm{C}-37{}^{\circ}\mathrm{C}\right),& T>100{}^{\circ}\mathrm{C}\end{array}\right\} $$

Finally, for the cell viability Ω, relating the robustness of each tissue to thermal damage, the temperature, and exposure time, the Arrhenius damage model [24] was utilised:

8 $$ \Omega (t)={\int}_0^t{\boldsymbol{Ae}}^{-\frac{\Delta \boldsymbol{E}}{\boldsymbol{RT}\left(\tau \right)}}d\boldsymbol{\tau}, $$

where A, a frequency factor, and ∆*E*, the activation energy for irreversible damage reaction, are cell-line-dependent parameters, and *R* is the universal gas constant. Because most of the thermal damage occurs in bone, osteocytes were chosen for the cell-line parameters, with values of *A* = 8.99 *x* 10^133^*s*^−1^ and ∆*E* = 838 kJ/mol [25]. Additionally, to assess the thermal damage and measure the extension of the ablation zone, a value of *Ω* = 4.6, corresponding to a 99% probability of cell death, was chosen as threshold, which was also the threshold to stop blood perfusion in Eq. (5).

### Boundary conditions

**Fig. 1:**
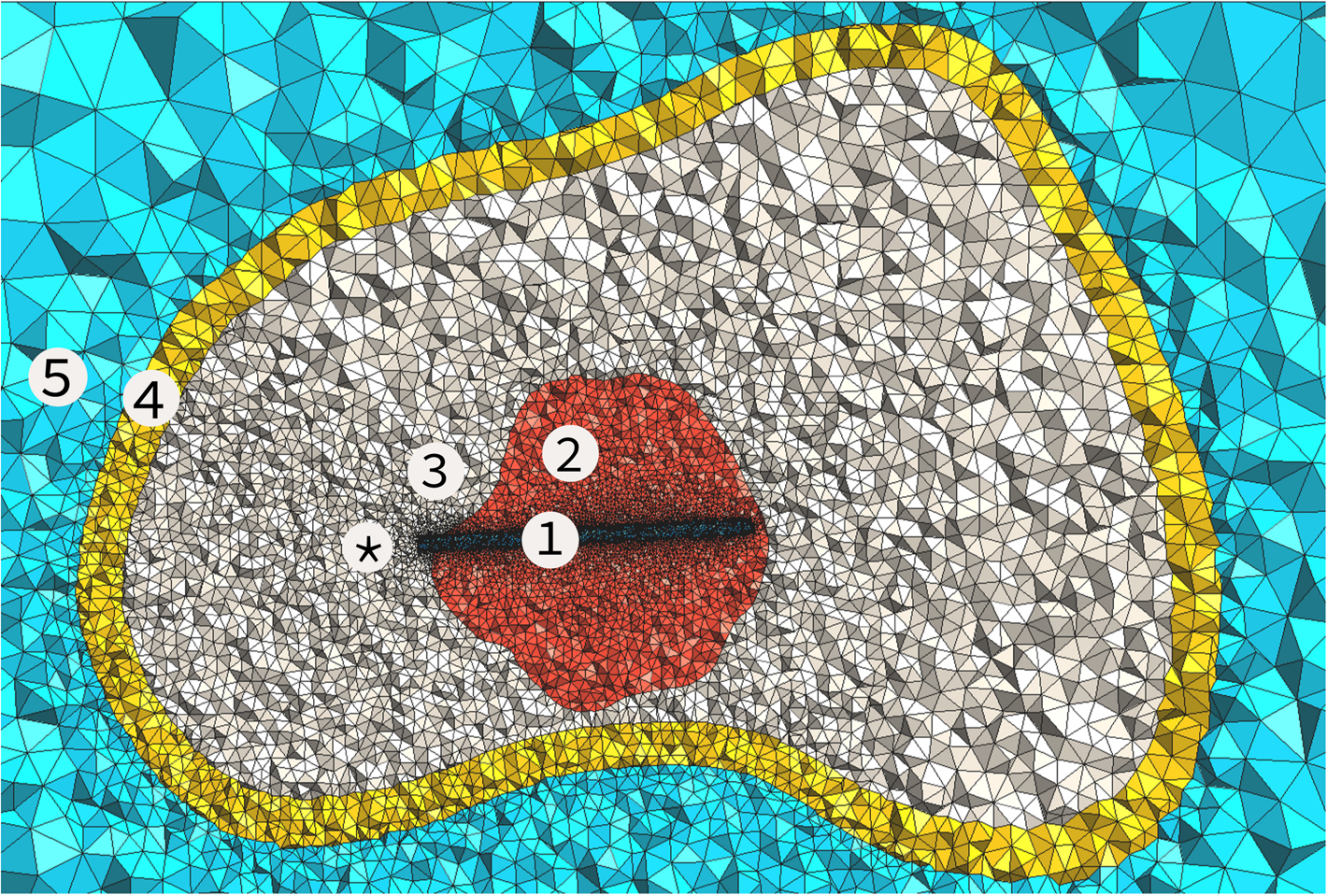
Cross-sectional view of one of the cases. The numbers indicate (1) the electrode, (2) tumour, (3) trabecular bone, (4) cortical bone, and (5) muscle. Boundary conditions were applied on the surface of the electrode and the outer surface of the muscle layer. *Indicates the bottom surface of the electrode was thermal and electrical no-flux boundary conditions were applied

A Dirichlet boundary condition [26] corresponding to the body temperature (37 °C) and one of zero voltage corresponding to the grounding pad were set at the outer boundaries of the muscle tissue for the thermal problem and electrical problems, respectively. At the electrode surface, a Dirichlet boundary condition equal to the applied RMS voltage was also set, which varies on time as defined by the proportional-integral controller. At the bottom side of the electrode, however, a Neumann boundary condition of zero was set for both the thermal and electrical problems, as that side is, in practice, part of a larger insulated piece with no heat or electrical flux. The insulated section of the electrode, which comes from the outside of the patient to the target site, was not modeled to simplify the 3D meshes. A cross section of one of the resulting meshes and the different tissues is shown in Fig. 1.

### Tissue properties

In a previous and yet to be published by our research group, optimal values tissues’ properties were found by taking the average values of electrical conductivity, blood perfusion, density, specific heat, and thermal conductivity of each tissue. For the tumour and its immediate surrounding tissue, trabecular bone, a special consideration was taken, where their values were varied within the maximum and minimum ranges reported in order to find the best fitting values. Additionally, for tumour tissue (ACT), the values from healthy cartilage were taken, except for the electrical conductivity, were twice the value of the electrical conductivity of healthy cartilage was needed in order to replicate the clinical cases, which was assumed based on the fact that multiple tumourous tissues have electrical conductivities higher than twice that of their healthy counterparts [27–29]. With all other properties kept at their average reported values, multiple combinations of the values for tumour and trabecular bone were tested. Using a fractional factorial model to avoid a full factorial analysis, the Taguchi method, with an array with ten variables and three levels (the five tissue properties of tumour and trabecular bone with their minimum, average, and maximum values), was utilised. A simplified 2D axisymmetric FEM model was utilised for this, replicating a simplified version of an actual clinical case, until the resulting ablated zone from the simulations was within acceptable range from the clinical results. The resulting optimised parameters replicated the ablated zone in the direction along the electrode, but slightly over-estimated the results on the direction perpendicular to it. These resulting parameters were tested in another 2D axisymmetric model of a different patient with similar results. The resulting values are shown in Table 1.

**Table 1:** Tissue and material properties

Material/tissue	Electrical conductivity (S/m)	Blood perfusion coefficient (x 10^***−*3**^***s***^***−*1**^)	Density (kg/m^3^)	Specific heat (J/kg ∙ K)	Thermal conductivity (W/m/K)
Active electrode ^a^	1.00 R + 08	0	6,450^a^	840	18
Tumour	0.4 ^b^	0	1,150	3,664	0.487
Trabecular bone	0.05	0.167	1,080	2,060	0.36
Cortical bone	0.022	0.167	1,908	1,313	0.32
Muscle	0.446	.6167	1,090	3,421	0.49

All values were obtained from [30], except where indicated. ^a^Values as described in [31], which are of a nickel-titanium electrode, ^b^assumptions for the electrical conductivity of the tumour, which are twice the value of normal healthy cartilage, also obtained from [30]

### Ablation time

The radiologist who performed the RFA procedure reported the ablation time in two ways: time ≥ 60 °C and time at 90 °C. The most important duration is the time at 90 °C, which is the desired target temperature for the procedure. Since the interventional radiologist may have difficulties reaching the target temperature, and because 60 °C is seen as an important threshold for tissue damage, both of these durations were reported to highlight the time needed to reach the target temperature and the time since thermal damage was certain to be happening. To replicate the cases, the simulated proportional-integral controller was set to 60 °C, and once reached, the target temperature was linearly increased depending on the time difference between both reported durations and the remaining 30 °C needed to reach 90 °C, after which it was finally set to 90 °C for the time indicated by the interventional radiologist as time at 90 °C.

## Results

**Fig. 2:**
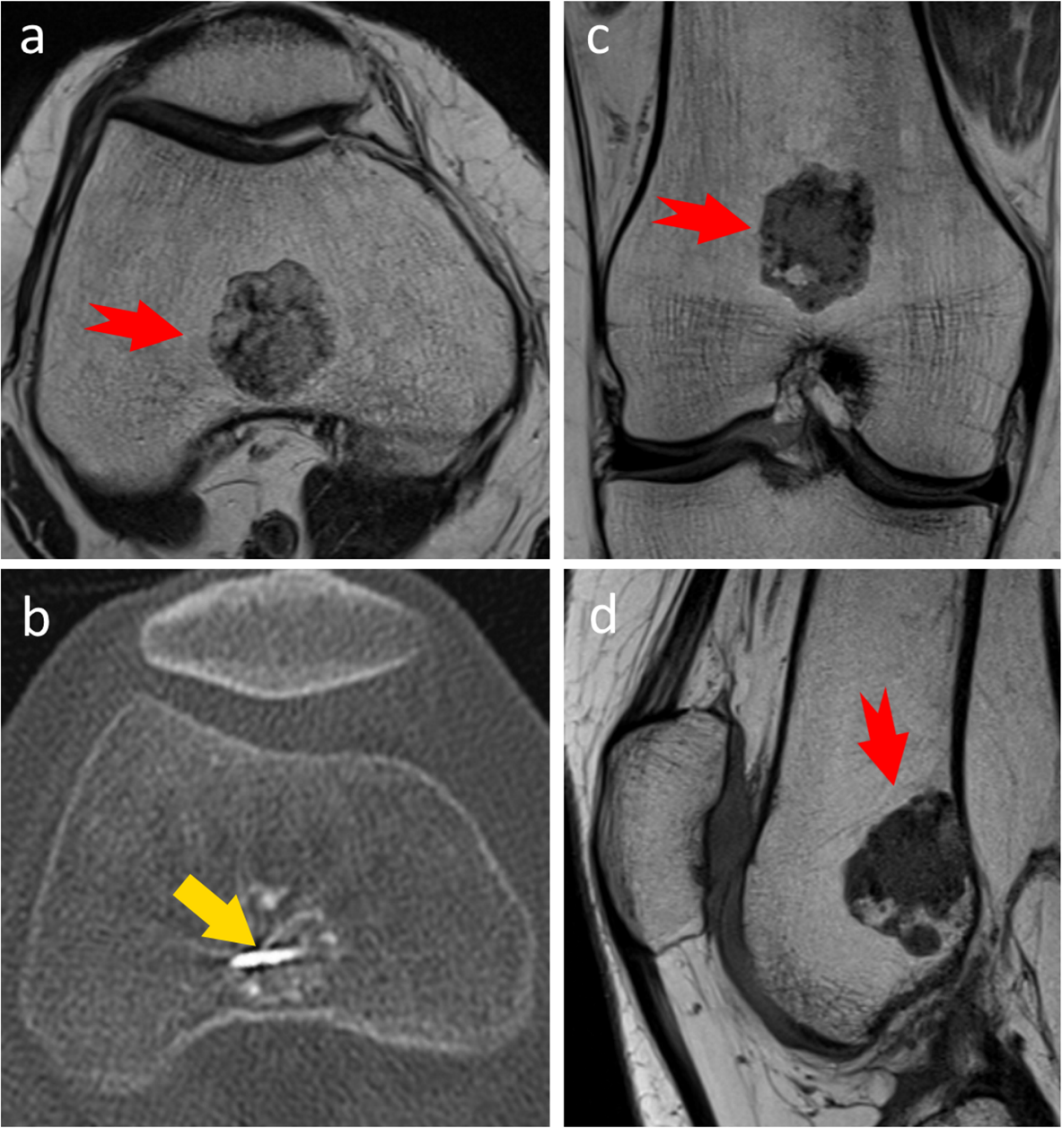
**a**, **c**, and **d** Preoperative magnetic resonance images in different planes to evaluate an atypical cartilaginous tumour located in the metaphysis of the distal femur, with the tumour being indicated by the red notched arrow. **b** An intraprocedural computed tomography image demonstrating the final location of the tip of the radiofrequency ablation electrode

**Fig. 3:**
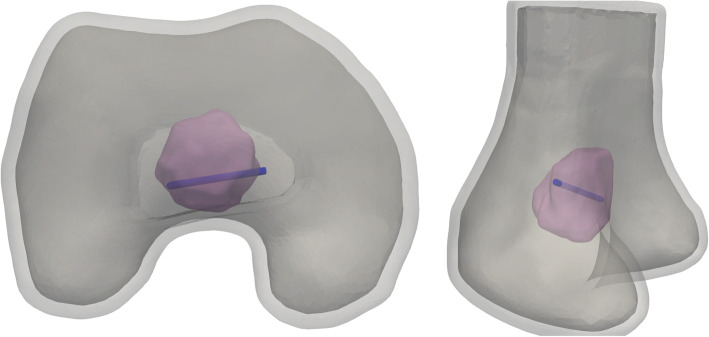
Example of the resulting three-dimensional models from the image segmentations. The electrode is shown in blue, the tumour in pink, and the bones in different shades of grey. The model corresponds to the case shown in Fig. 1

From the fifteen patient cases available, seven cases were found where the final position of the electrode was visible and the imaging data allowed for good segmentation and registration of the MR and CT images. Eight cases were excluded because the final position of the electrode was missing or because of a limited field of view or resolution that did not allow for a good image segmentation or registration. Figure 2 shows the pre- and intra-operative images of one of the cases, indicating the tumour and electrode, whereas Fig. 3 shows the resulting image segmentation of the same case.

**Fig. 4:**
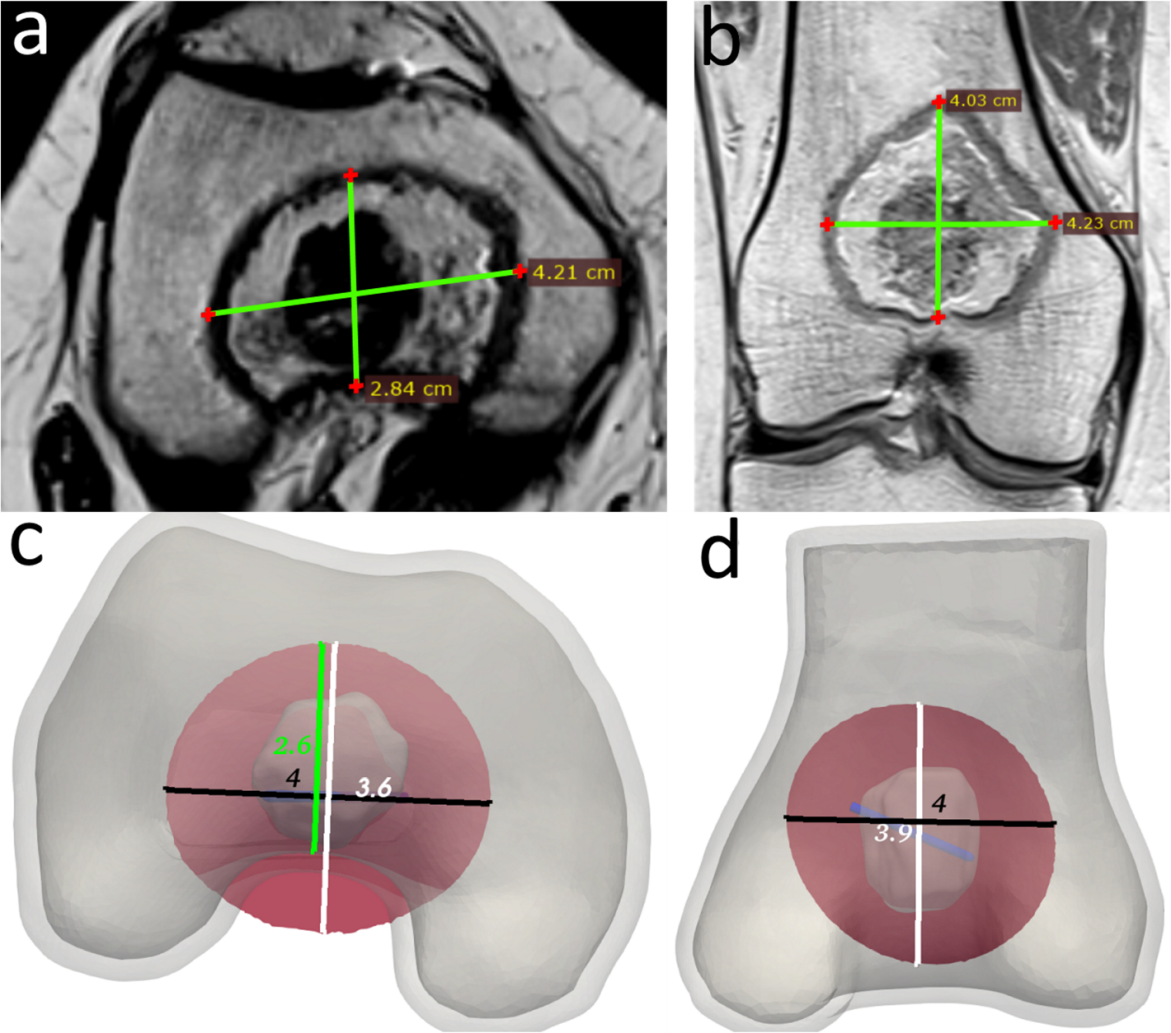
**a**, **b** Resulting ablation diameters on the magnetic resonance imaging (MRI) study performed 3 months after the radiofrequency ablation (the same case of Figs. 2 and 3). **c**, **d** Resulting ablation diameters from the simulations. The ablation zones are represented as a transversal cut at the height of the electrode, inside the three-dimensional geometry, to match the direction of the MRI measurements. **c** The green line is the measurement of the ablation diameter as if it were to be taken until the cortical bone and is shown here as an example of the main difference between the results from the simulations and the radiological measurements

The ablation diameter on the post-RFA images seemed to stop at the boundary with the cortical bone. However, this was not observed in the computer simulations, where the ablation radius extended beyond the cortical bone, as seen in Fig. 4 a, c. The reported ablation radius from the simulations was the full diameter beyond the cortical bone, and this was the source of the largest discrepancies between the simulations and the radiological measurements, except for case number two, where there was an unexpectedly large ablation diameter along the electrode.

**Fig. 5:**
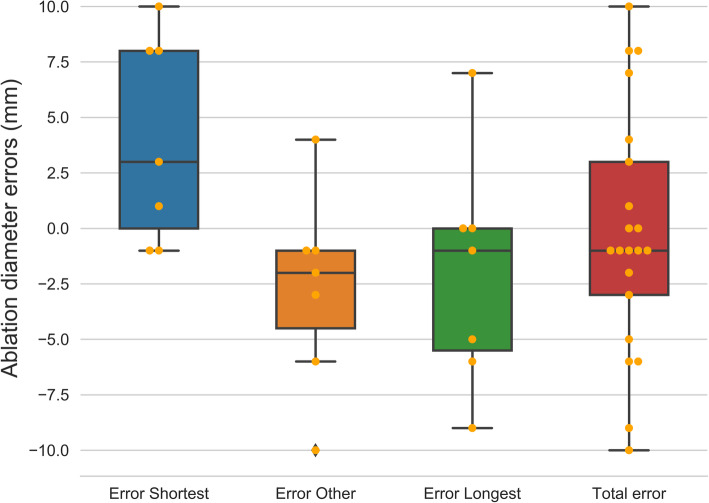
Boxplots of the errors of the resulting diameters for the three directions measured and the “total error” taking into account every measurement. All values are in millimeter

A total median error of -1 mm was achieved with interquartile ranges from -3 to 3 mm. The ablation diameters for the radiological images and computer models are shown in Table 2. The error, calculated as the radiological measurements minus the results from the computer models, is presented in Table 3 and shown as boxplots in Fig. 5. The computer simulations overestimated the shortest ablation diameter in most of the cases and underestimated it in the other two directions.

**Table 2:** Cases, length of the electrode used, timings, and the resulting ablation diameters from the radiological measurements and the simulations

Case	Electrode length (cm)	Time (min) > 60 °C	Time (min) at 90 °C	Ablation shortest (mm)	Ablation other (mm)	Ablation longest (mm)	Simulation shortest (mm)	Simulation other (mm)	Simulation longest (mm)
1	2	12	10	26	40	42	36	39	41
2	3	10	8	41	52	63	42	42	54
3	3	10	6	28	39	48	36	36	48
4	2	10	9	40	42	43	39	40	43
5	2	9	9	43	48	51	42	42	45
6	3	10	8	37	34	46	45	38	41
7	2	10	9	24	30	30	27	29	37

**Table 3:** Absolute (in mm) and relative (as percentage, deviating from 100%) errors, including the median and interquartile ranges (IQR) per measured direction, and the total error taking every measurement into account

Case	Error shortest (mm)/percentage		Error other (mm)/percentage		Error longest (mm)/percentage		
1	10	138%	-1	98%	-1	98%	
2	1	102%	-10	81%	-9	86%	
3	8	129%	-3	92%	0	100%	
4	-1	98%	-2	95%	0	100%	
5	-1	98%	-6	88%	-6	88%	
6	8	122%	4	112%	-5	89%	
7	3	113%	-1	97%	7	123%	
Error interquartile ranges (IQR)							Total error
IQR1 (mm)	0		-4.5		-5.5		−3
Median (mm)	3		-2		-1		-1
IQR3 (mm)	8		-1		0		3

**Fig. 6:**
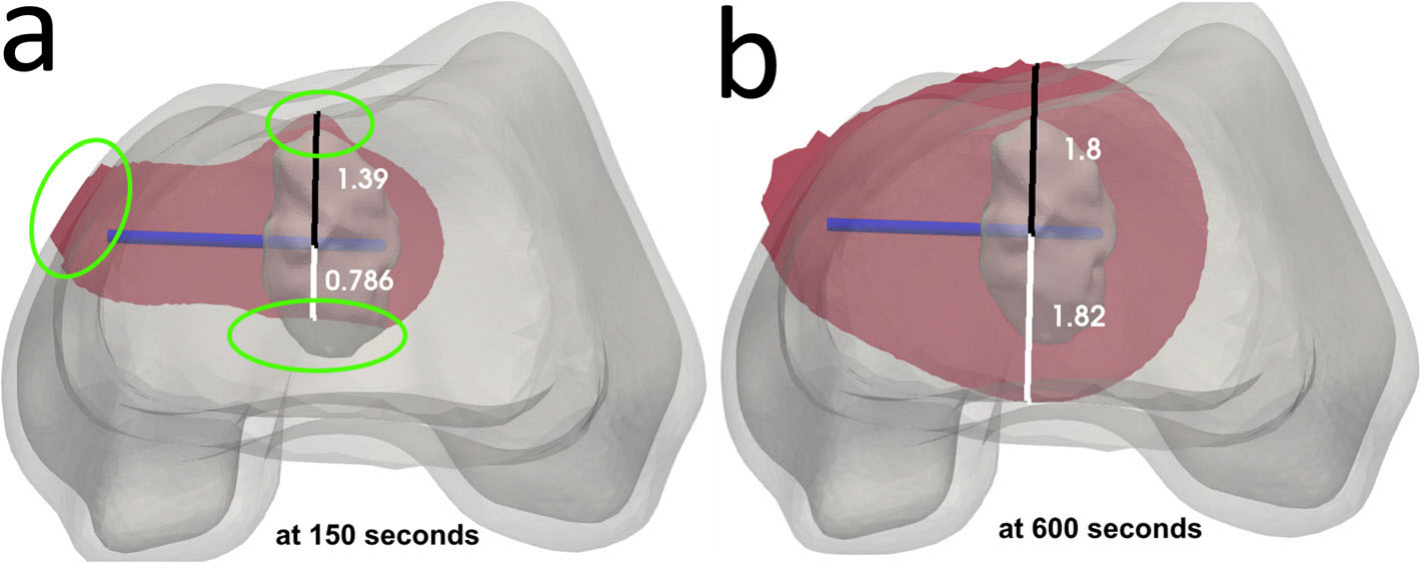
Progression of the ablation zone of case three with an atypical cartilaginous tumour in the distal femoral metaphysis at two selected times. Both ablation zones are represented as a transversal cut at the height of the electrode, inside the three-dimensional geometry. **a** The ablation zone after 150 s, when the ablation zone is still rapidly growing. **b** The ablation zone at the end of the procedure after steady state has been reached. The measurements were taken from the centre of the electrode and perpendicular to it. All measurements are in centimeter

The effect of the more complex 3D anatomy was particularly noticeable in the early stages of the ablation procedure but less so towards the end. After the procedure had reached steady state, as shown in Fig. 6a, heat concentrated close to the cortex causing damage more quickly in that direction. However, as time passed, the thermal damage became more evenly distributed, as shown in Fig. 6b.

## Discussion

This study demonstrated that patient-specific simulations of RFA of bone tumours could potentially be used to predict the resulting ablation zone. Although the number of patients was relatively limited in our series, with a median error of < 3 mm in all directions measured, the tissue properties utilised in the simulations seem to be accurate enough to predict the outcomes within a reasonable range and could potentially be used for patient planning.

The simulations tended to produce uniform ablation zones, with the largest ablation diameter in the direction of the longitudinal axis of the electrode, and with an ablation diameter that was roughly uniform in length in all perpendicular directions measured. In contrast, patient data showed more variations, which could also be due to anisotropies in the bone that were not taken into account in our models. On the clinical post-RFA MRI scans, ablation zones did not seem to extend beyond the bone in most cases, but our simulations did not show this effect. In fact, when the tumour was next to the cortical bone, heat seemed to concentrate in that area. This effect was, however, not noticeable in the steady state of the system after several minutes had passed, where the ablation zone was more evenly distributed.

Given the slow rate at which the bone heals in contrast to the muscle and that the post-RFA MR images were taken approximately 3 months after the procedure, it is possible that the damage to the muscle may not have been visible anymore. Lee et al. performed RFA in the distal healthy femurs of seven dogs, showing that MR images could be reliably used to measure the extent of the ablation zone and that the resulting ablation zones clearly extended beyond the cortical bone, as shown on contrast-enhanced fat-suppressed T1-weighted images taken 4 to 7 days after the procedure [32]. Thus, it is possible that the apparent overestimation in our simulations, with the ablation zones extending beyond the cortex, could be much more accurate than it seems. This difference was particularly remarkable in cases 1, 3, and 8, which were tumours close to a cortical wall and which had errors of 10, 8, and 8 mm, respectively, in the “shortest direction,” whereas the errors were small when the simulated ablation zone did not extend significantly beyond the cortical wall.

Another issue of our study was the relatively low resolution of the MR images. The original idea was to compare the volumes of the segmentation on post-RFA MR images to those of the simulations, but this proved to be very imprecise due to the large MR slice thickness (5 mm), making the segmentation of the ablation zone very difficult and imprecise at the boundaries, particularly because of the diffuse ring that defines it. These errors compound when taking all directions into account as in the 3D segmentations, which could result in large differences in volume. Because of this, we opted to report and compare the diameters rather than the volumes. Moreover, the ablation diameters are what radiologists usually report and clinicians use for follow-up purposes.

Another source of uncertainty came from the reported durations of the procedure, as in case number five, which stated identical durations at temperature ≥ 60 °C and at 90 °C, and thus, it was not clear what the time was to reach from 60 to 90 °C. The durations where not reported in detail, and some assumptions had to be done. Additionally, the electrode could have been moved during the intervention, and the final CT scan taken may not represent the actual final position of the electrode. Given how much both the procedural time and the position of the electrode affect the ablation zone, this may have affected the predicted size of the ablation zone. In case number two, for example, where the resulting ablation zone was considerably larger even when compared to cases with almost identical parameters (like case number 6), it seems possible that the large error in the unusually large ablation zone may be due to a repositioning of the electrode that was not registered and thus also resulting in a large error when compared to our simulations.

There are currently no guidelines to predict the resulting ablation zone in RFA of ACT in the long bones, which are needed for accurate and safe patient planning. Our results seem to indicate that, if planned prospectively, with an accurate position of the electrode and exact duration of the procedure, computer simulations could predict the ablation diameters within a reasonable accuracy and be thus utilised for patient planning, as shown in our results, where a total median error of -1 mm was achieved. The tissue properties of the tumours have not been studied yet, but this study demonstrated that they could be reverse-engineered within reasonable accuracy for planning, and they could be made more reliable with more patient data to fit the loss function. This could allow interventional radiologists to accurately define the time, electrode length, and position required to treat ACT with RFA and make adjustments as needed to guarantee total tumour destruction while sparing as much healthy tissues as possible.

## Data Availability

The data supporting the findings of this study are available within the article, and raw data from the computer simulations are available from the corresponding author on request.

## Abbreviations

2D: Two-dimensional
3D: Three-dimensional
ACT: Atypical cartilaginous tumour
CT: Computed tomography
FEM: Finite element method
MR: Magnetic resonance
RFA: Radiofrequency ablation
RMS: Root mean square
STL: Standard triangle language
